# Broadband Spin-Dependent Directional Coupler via Single Optimized Metallic Catenary Antenna

**DOI:** 10.3390/ma14020326

**Published:** 2021-01-10

**Authors:** Cong Chen, Jiajia Mi, Panpan Chen, Xiang Du, Jianxin Xi, Li Liang, Jianping Shi

**Affiliations:** 1 College of Physics and Electronic Technology, Anhui Normal University, Wuhu 241000, China; congchen199618@163.com (C.C.); 2015029@ahnu.edu.cn (J.M.); chenpanpan@ahnu.edu.cn (P.C.); xiangdu@ahnu.edu.cn (X.D.); xijianxin@ahnu.edu.cn (J.X.); 2 Anhui Province Key Laboratory of Photo-Electronic Materials Science and Technology, Wuhu 241000, China

**Keywords:** sub-wavelength structure, SOI waveguide, broadband, spin-dependent

## Abstract

With the rapid development of on-chip optics, integrated optical devices with better performance are desirable. Waveguide couplers are the typical integrated optical devices, allowing for the fast transmission and conversion of optical signals in a broad working band. However, traditional waveguide couplers are limited by the narrow operation band to couple the spatial light into the chip and the fixed unidirectional transmission of light flow. Furthermore, most of the couplers only realize unidirectional transmission under the illumination of the linear polarized light. In this work, a broadband polarization directional coupler based on a metallic catenary antenna integrated on a silicon-on-insulator (SOI) waveguide has been designed and demonstrated under the illumination of the circularly polarized light. By applying the genetic algorithm to optimize the multiple widths of the metallic catenary antenna, the numerical simulation results show that the extinction ratio of the coupler can be maintained larger than 18 dB in a wide operation band of 300 nm (from 1400 to 1700 nm). Moreover, the coupler can couple the spatial beam into the plane and transmit in the opposite direction by modulating the rotation direction of the incident light. The broadband polarization directional coupler might have great potential in integrated optoelectronic devices and on-chip optical devices.

## 1. Introduction

With the development of subwavelength optical engineering [1,2,3,4,5,6], the integrated optics have shown great application prospects in the fields of super-resolution imaging [7,8], nanolithography [5,9,10,11], surface plasmon excitation [12,13,14,15,16], extraordinary Young’s interference experiment [17,18], and meta-surface-wave [19,20,21]. Improving the transmission efficiency of couplers is becoming more and more important [22,23,24,25,26,27,28,29,30].

Recently, a new type of waveguide coupler [31,32,33] for electromagnetic waves has been designed by combining surface plasmon polaritons (SPPs) and optical silicon waveguides, which have strong plasma confinement characteristics and retain low transmission loss property of the waveguide [34,35]. However, the majority of reported directional waveguide couplers can only work under the irradiation of linearly polarized light. Furthermore, the transmission direction of the couplers cannot be adjusted. Fortunately, the above limitations can be effectively solved by the geometric phase metasurface. For example, a controlled directional router can be realized by placing seven anisotropic antennas on the silicon-on-insulator (SOI) waveguide [36] due to the geometric phase metasurface [37,38]. With further in-depth research on subwavelength structures, catenary optics has been proposed and developed in recent years. Catenary optics points out that the catenary structure of sub-wavelength size possesses geometric phase modulation ability due to the spin-orbit interaction [4,38,39,40]. In 2018, a waveguide coupler based on a single metallic catenary structure had been proposed which can adjust the optical transmission direction by reversing the spin of the incident light [41].

Although the above studies have enriched the working polarization and controlled the light flow, the couplers still have a conspicuous shortcoming of the narrow bandwidth, which leads to the inconvenience of practical use. In this work, we managed to broaden the bandwidth by resorting to genetic algorithms (GA) to optimize the multiple widths of a single metallic catenary antenna. The built-in GA framework in MATLAB drives the FDTD software to obtain an optimal catenary structure. The new coupler designed with optimal parameters has a working bandwidth of 300 nm (ranging from 1400 to 1700 nm) for the extinction ratio remaining higher than 18 dB. Besides, the coupler retains the response to the rotation of the incident light, and the transmission direction can be changed by reversing the spin of the incident light. The broadband characteristics of this device would have great potential in plasma integrated circuits.

## 2. Design Principles and Simulation Results

The catenary is a curved shape of a chain with uniform quality and soft fixed at both ends under the action of gravity. Since the catenary structure has the same load at each point, it is widely used in the design of building structures [42,43]. The formula for the catenary of equal strength was deduced by Gilbert [44]:

(1) $$y=\frac{\Lambda }{\pi }\ln\left(\left|\sec\left(\pi x/\Lambda \right)\right|\right),$$

**Figure 1:**
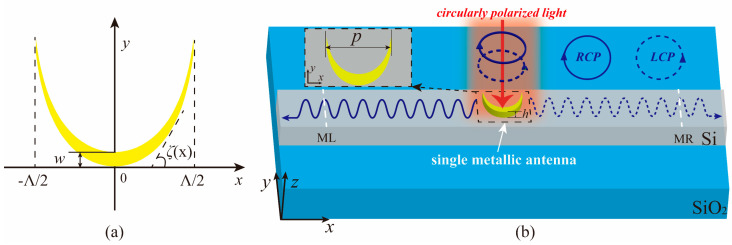
(**a**) Catenary of equal strength in a rectangular coordinate system. (**b**) The schematic of the directional coupler is composed of a single metallic catenary antenna placed on a silicon-on-insulator (SOI) waveguide. The white dotted lines indicate two power monitor ML and MR, and the monitors are 10 μm away from the antenna.

(2) y1=y+w,

where Λ is the length of the catenary in the horizontal direction. The range of ***x*** is between −Λ/2 and +Λ/2, because the value of y is infinite for *x* = ± Λ/2 according to Equation (1). Moreover, a truncation factor *f* is introduced to represent the actual horizontal span of the catenary as *p* = *f*Λ. Equation (2) is obtained after Equation (2) is translated *w* in the +*y* direction. As shown in Figure 1a, we can draw the catenary structure in the rectangular coordinate system according to Equations (1) and (2). It is worth noting that the structure is composed of two catenary curves of the identical shape connected. Here, the amount ***w*** of translation in Equation (2) represents the vertical distance between the two curves of the catenary structure.

To study catenary in more detail, we took the derivative of the formula for the catenary of equal strength:

(3) $$y^{\prime }=\tan\frac{\pi x}{\Lambda }$$

Figure 1a shows that ζ(*x*) is the angle between the tangent of the catenary and the positive direction of the x-axis. Based on the meaning of the tangent angle of the curve and Equation (3), the expression of ζ(*x*) can be derived:

(4) $$\zeta (x)=\arctan(y^{\prime })=\frac{\pi x}{\Lambda },$$

Substituting the value of x into Equation (4), the value range of ζ(*x*) can be calculated, which varies from −π/2 (*x* = −Λ/2) to π/2 (*x* = +Λ/2). Considering the theory of geometric phase, the catenary can create a continuous geometric phase Φ(*x*) = 2σζ(*x*), where *σ* = +1 or *σ* = −1 represents left-handed circularly polarized light (LCP) and right-handed circularly polarized light (RCP), respectively. Combined with Equation (4), the catenary structure has a linear geometric phase, which can be described as

(5) $$\Phi (x)=\frac{2\sigma \pi x}{\Lambda },$$

Here, the linear phase gradient dΦ(*x*)/d*x* = 2*σ*π/Λ of the catenary can be calculated by Equation (5). According to the generalized Snell’s law [45,46,47], the sign of phase gradient determines the coupling direction, which is thus further determined by the value of *σ*. Consequently, the coupling direction of light flow can be changed by reversing the spin of incidence (LCP or RCP).

**Figure 2:**
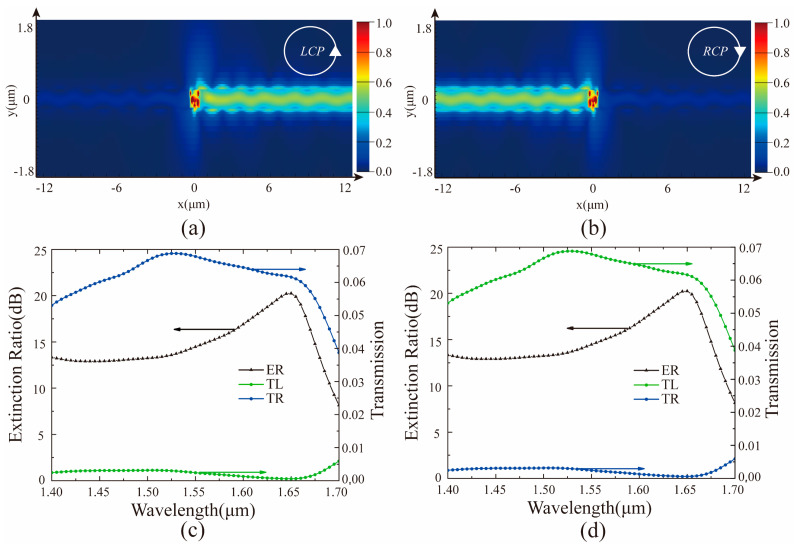
The electric field distribution with a wavelength of 1650 nm under the illumination of (**a**) left-handed circularly polarized light (LCP) and (**b**) right-handed circularly polarized light (RCP) light, respectively. The power transmittance of the two monitors (ML and MR) under the incidence of (**c**) LCP and (**d**) RCP light, respectively. TL represents the transmittance measured by the power monitor ML, and TR represents the transmittance measured by the power monitor MR.

To verify the characteristics of the catenary structure, we simulated a directional coupler composed of a single metallic catenary antenna placed on an SOI waveguide by using the FDTD Solution software (see Figure 1b). Here, the thickness and width of the silicon waveguide were 0.22 μm and 0.5 μm, respectively. The refractive index of silicon was taken from the data of Palik [48]. The thickness *h*, horizontal length Λ, and width ***w*** of the gold catenary antenna were set as 0.09 μm, 0.9 μm, 0.1 μm, respectively. Considering the limited width of the silicon waveguide, the truncation factor ***f*** of the catenary was set to 0.7. The relative permittivity setting of gold refers to the data of Johnson and Christy [49]. The refractive index of the substrate material SiO_2_ was from the data of Palik [48]. Furthermore, the boundary conditions were set as perfectly matched layer in all directions to avoid the influence of the boundary reflection. As shown in Figure 2, the simulation results were consistent with the theoretical prediction. The coupling direction of the beam was modulated by the rotation of the incident light. Specifically, Figure 2a shows that the light stream flows to the right under the LCP light. However, when the RCP light is irradiated, the light stream flows to the left as shown in Figure 2b.

**Figure 3:**
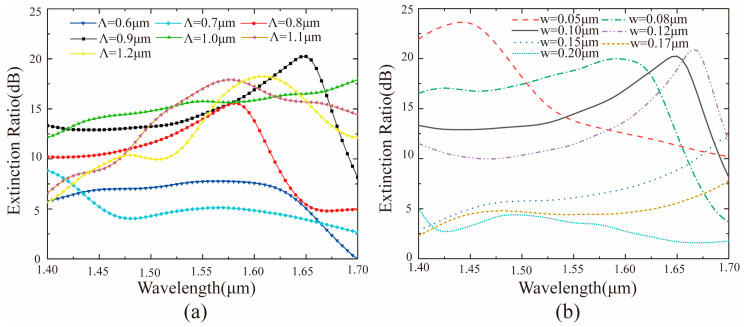
The relationship between the extinction ratio of the directional coupler and the corresponding wavelength, when changing the geometric parameters of the catenary structure. (**a**) Shifting the horizontal length Λ of the catenary. (**b**) Shifting the width w of catenary structure.

It is necessary to introduce the concept of extinction ratio (ER), which is defined as ER_RCP_ = 10 × log(TL/TR) or ER_LCP_ = 10 × log(TR/TL), to evaluate the performance of the coupler. Here, the value of the extinction ratio reflects the strength of directivity. Based on the results of the simulation in Figure 2c,d, it can be found that the bandwidth (ER > 18 dB) of the coupler is only about 30 nm, which is too narrow compared to the previously reported couplers [41]. However, the extinction ratio of the coupler also needs to be improved. To extend the working bandwidth and further improve the extinction ratio, the geometric parameters of the catenary structure have been fully optimized. Figure 3 shows that the extinction ratio and working bandwidth of the directional coupler are greatly affected by the geometric parameters of the catenary structure placed on the SOI waveguide. These findings laid the foundation for the design of a broadband coupler. After considering the extinction ratio and bandwidth (ER > 18 dB) in Figure 3a, the horizontal length Λ of the catenary structure was fixed to 0.9 μm to design a broadband polarization directional coupler with strong directionality. Moreover, the width w of the catenary structure has a more significant effect on the bandwidth of the coupler as shown in Figure 3b. Thus, the multiple widths of the catenary structure should be optimized at the same time to extend the bandwidth of the device.

**Figure 4:**
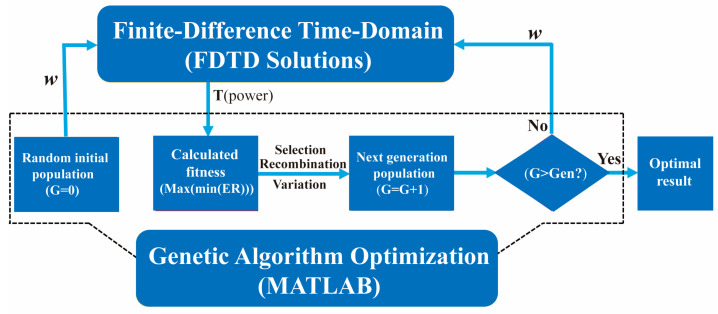
The flow chart of the genetic algorithm (GA) optimization simulation process based on MATLAB and FDTD Solutions.

Since it is inefficient to improve the performance of the coupler through full parameters scanning optimization based on FDTD Solutions software, we alternatively utilize optimization algorithms to shorten the cycle of structural optimization in the design. Among a lot of optimization algorithms, genetic algorithm (GA), which is widely used in the design of optical components [50,51,52], is selected to optimize the multiple widths of the catenary structure. The simulation software FDTD Solutions has provided an application programming interface to MATLAB so that we can run FDTD Solutions with MATLAB. Thus, MATLAB and FDTD Solutions software are used to realize joint simulation to optimize the multiple widths of the catenary structure, as shown in the flow chart in Figure 4.

**Figure 5:**
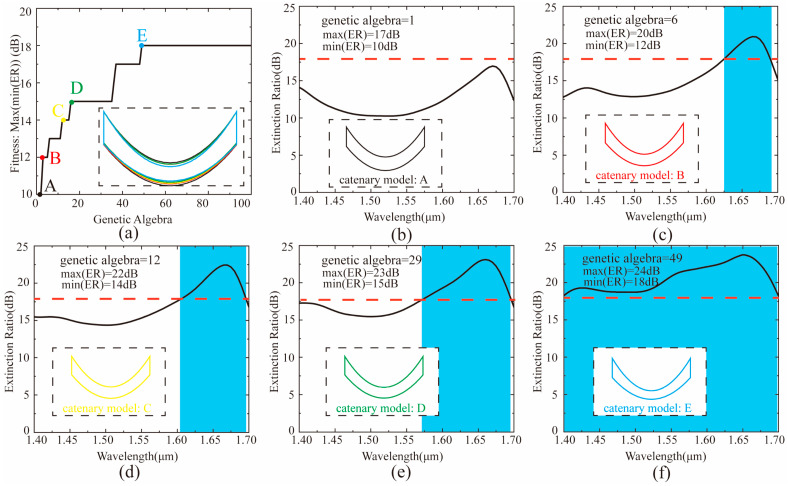
(**a**) The relationship between fitness (Max(min(ER))) function and genetic algebra. The illustration represents the optimal catenary structure at points A – E. (**b–f**) represent the relationship between the extinction ratio and the corresponding wavelength at points A – E in (**a**), respectively. The red dotted lines indicate that the extinction ratio is equal to 18 dB. The blue areas indicate the working bandwidth range when the extinction ratio is greater than 18 dB.

According to the optimized process, the GA program framework built in MATLAB drives FDTD Solutions, and realizes the data exchange between the MATLAB and FDTD Solutions. Here, the population size was set to 30, and the genetic generation number was set to 100. Taking into account the width limitation of the silicon waveguide in the coupler, we set the upper and lower limits of the catenary structure width to be 0.05 μm and 0.2 μm, respectively. Then, the coordinate information of catenary structures with different widths was transferred to FDTD Solutions. Based on these coordinate data, some FDTD files with catenary structures of different widths were established by using the scripting language. After running these FDTD files, we calculated the extinction ratio with respect to the wavelength through obtained transmittance from two power monitors (ML and MR). Next, the minimum extinction ratio of all FDTD files was fed back to MATLAB. Finally, in order to select the optimal catenary structure in each generation, we defined a fitness function (Max(min(ER))), which represents the maximum value of the minimum extinction ratio of all populations in the generation. Figure 5a shows that the value of Max(min(ER)) gradually increases as the genetic algebra increases. However, when the genetic algebra increases to 49, the value of Max(min(ER)) remains unchanged. In other words, the optimal catenary structure is obtained when the genetic algebra is 49. To specifically describe the increase in the bandwidth of the coupling device during the optimization process, we selected five optimal catenary structures of the genetic algebra (corresponding to point A to Point E) during the optimization process from Figure 5a for simulation. Based on the simulation results, it is obvious that the bandwidth (ER > 18 dB) of the coupler in Figure 5b–f widens as the genetic algebra increases.

**Figure 6:**
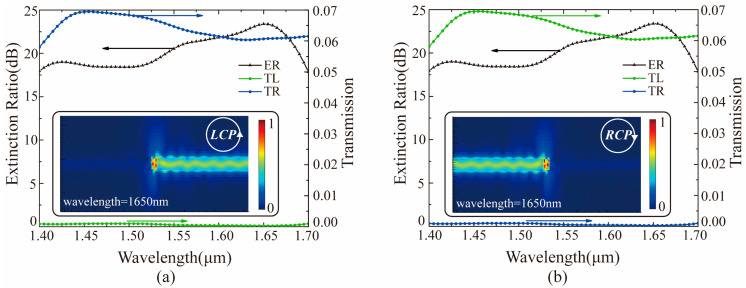
The extinction ratio and power transmittance of the directional coupler is optimized by GA under the incidence of circularly polarized light. The insets show the electric field distribution in the silicon waveguide at the wavelength of 1650 nm. (**a**) The illustration of LCP. (**b**) The illustration of RCP.

**Figure 7:**
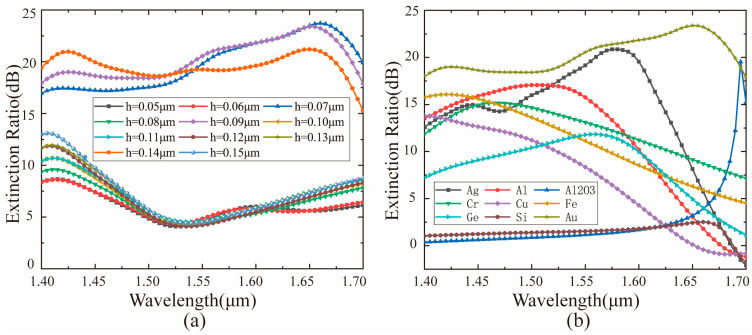
The influence of catenary antenna thickness and material on extinction ratio and operating bandwidth of the coupler. (**a**) The thickness values of the catenary antenna are different. (**b**) Catenary antennas are composed of different materials.

By optimizing the width of the catenary using GA, the bandwidth of the coupler has been broadened. As indicated in Figure 6, the bandwidth of the designed coupler increases to 300 nm where the extinction ratio remains greater than 18 dB. Compared to the previous research [41], the bandwidth increases by approximately three times, and the power transmittance of the transmission side has also been improved. Also, it is found that the GA optimization does not affect the symmetry of the catenary structure according to the two insets in Figure 6. We can still control the directions of light flow by shifting the spins of the incident light. Furthermore, we analyzed the influence of thickness and materials of the catenary antenna on the extinction ratio and bandwidth of the coupler. As shown in Figure 7a, the extinction ratio and working bandwidth of the coupler are sensitive to the thickness of the catenary antenna. Specifically, the coupler has a bandwidth larger than 18 dB when the thickness of catenary antennas is 0.07 μm, 0.09 μm, and 0.14 μm. The bandwidth (ER > 18 dB) of the coupler can reach 300 nm only if the thickness of the catenary antenna is 0.09 μm. Figure 7b shows that the materials of the catenary antenna have a sufficient effect on the extinction ratio and working bandwidth of the coupler. It is worth noting that metallic materials can promote the coupler’s performance compared to dielectric materials. The reason for this phenomenon is that SPPs are more easily excited by light illuminating on the metallic surface.

## 3. Conclusions

This research uses genetic algorithms (GA) to optimize the multiple widths of a single metallic catenary antenna integrated on the SOI waveguide to design and demonstrate the broadband polarized directional coupler under the incidence of circularly polarized light. Numerical simulation results show that the extinction ratio of the coupler can be maintained above 18 dB in a broad band (300 nm, ranging from 1400 nm to 1700 nm). Moreover, because the symmetry of the catenary structure is preserved in the optimization process using GA, the coupler can not only couple the spatial beam into the plane, but also reverse the light flow by modulating the spin of the incidence. The broadband polarization directional coupler would have great application potentials in integrated optoelectronic devices and on-chip optical communications.

## Data Availability

The data that support the findings of this study are available within the article.

## References

[1] Guo J., Wang T., Quan B., Zhao H., Gu C., Li J., Wang X., Situ G., Zhang Y. (2019). Polarization multiplexing for double images display. Opto-Electron. Adv..

[2] Luo X. (2018). Subwavelength optical engineering with metasurface waves. Adv. Opt. Mater..

[3] Luo X. (2018). Engineering optics 2.0: A revolution in optical materials, devices, and systems. ACS Photonics.

[4] Luo X. (2019). Catenary Optics.

[5] Luo X., Ishihara T. (2004). Surface plasmon resonant interference nanolithography technique. Appl. Phys. Lett..

[6] Sun S., He Q., Hao J., Xiao S., Zhou L. (2019). Electromagnetic metasurfaces: Physics and applications. Adv. Opt. Photonics.

[7] Smolyaninov I.I., Hung Y.-J., Davis C.C. (2007). Magnifying superlens in the visible frequency range. Science.

[8] Zeng B., Yang X., Wang C., Feng Q., Luo X. (2010). Super-resolution imaging at different wavelengths by using a one-dimensional metamaterial structure. J. Opt..

[9] Ebbesen T.W., Lezec H.J., Ghaemi H., Thio T., Wolff P.A. (1998). Extraordinary optical transmission through sub-wavelength hole arrays. Nature.

[10] Liu Z.-W., Wei Q.-H., Zhang X. (2005). Surface plasmon interference nanolithography. Nano Lett..

[11] Luo X. (2019). Engineering Optics 2.0: A Revolution in Optical Theories, Materials, Devices and Systems.

[12] Chen C., Chen P., Xi J., Huang W., Li K., Liang L., Shi F., Shi J. (2020). On-chip monolithic wide-angle field-of-view metalens based on quadratic phase profile. AIP Adv..

[13] Guo Y., Zhang Z., Pu M., Huang Y., Li X., Ma X., Xu M., Luo X. (2019). Spoof plasmonic metasurfaces with catenary dispersion for two-dimensional wide-angle focusing and imaging. Iscience.

[14] Liu Y., Palomba S., Park Y., Zentgraf T., Yin X., Zhang X. (2012). Compact magnetic antennas for directional excitation of surface plasmons. Nano Lett..

[15] Xu T., Zhao Y., Gan D., Wang C., Du C., Luo X. (2008). Directional excitation of surface plasmons with subwavelength slits. Appl. Phys. Lett..

[16] Chen P., Chen C., Qin S., Xi J., Huang W., Shi F., Li K., Liang L., Shi J. (2020). Efficient planar plasmonic directional launching of linearly polarized light in a catenary metasurface. Phys. Chem. Chem. Phys..

[17] Luo X., Pu M., Li X., Guo Y., Ma X. (2019). Young’s double-slit interference enabled by surface plasmon polaritons: A review. J. Phys. D.

[18] Pu M., Guo Y., Li X., Ma X., Luo X. (2018). Revisitation of extraordinary young’s interference: From catenary optical fields to spin–orbit interaction in metasurfaces. ACS Photonics.

[19] Luo X., Astronomy (2015). Principles of electromagnetic waves in metasurfaces. Sci. China: Phys. Mech. Astron..

[20] Luo X., Pu M., Guo Y., Li X., Zhang F., Ma X. (2020). Catenary Functions Meet Electromagnetic Waves: Opportunities and Promises. Adv. Opt. Mater..

[21] Pu M., Ma X., Guo Y., Li X., Luo X. (2018). Theory of microscopic meta-surface waves based on catenary optical fields and dispersion. Opt. Express.

[22] Bao Y., Liang H., Liao H., Li Z., Sun C., Chen J., Gong Q. (2017). Efficient unidirectional launching of surface plasmons by multi-groove structures. Plasmonics.

[23] Barnes W.L., Dereux A., Ebbesen T.W. (2003). Surface plasmon subwavelength optics. Nature.

[24] Huang S., Wang C.-Y., Chen H.-Y., Lin M.-H., Lu Y.-J., Gwo S. (2016). Dual-band planar plasmonic unidirectional launching in a semiannular apertures array. ACS Photonics.

[25] Huang W., Yang J., Xiao X., Zhang J. (2015). Surface Plasmon Polariton Unidirectional Nano-Launcher Based on the Strong Coupling Effects in an Asymmetric Optical Slot Nanoantenna Pair. Plasmonics.

[26] Liu D., Sun L., Lu F., Xu A. (2017). Ultrabroadband and wide-angle unidirectional coupling of surface plasmons based on chirped-nanoslits grating. J. Lightwave Technol..

[27] López-Tejeira F., Rodrigo S.G., Martín-Moreno L., García-Vidal F.J., Devaux E., Ebbesen T.W., Krenn J.R., Radko I., Bozhevolnyi S.I., González M.U. (2007). Efficient unidirectional nanoslit couplers for surface plasmons. Nat. Phys..

[28] Ritchie R.H. (1957). Plasma losses by fast electrons in thin films. Phys. Rev..

[29] Sun C., Chen J., Yao W., Li H., Gong Q. (2015). Manipulating surface-plasmon-polariton launching with quasi-cylindrical waves. Sci. Rep..

[30] Zayats A.V., Smolyaninov I.I., Maradudin A.A. (2005). Nano-optics of surface plasmon polaritons. Phys. Rep..

[31] Bernal Arango F., Kwadrin A., Koenderink A.F. (2012). Plasmonic antennas hybridized with dielectric waveguides. ACS Nano.

[32] Sidiropoulos T.P., Nielsen M.P., Roschuk T.R., Zayats A.V., Maier S.A., Oulton R.F. (2014). Compact optical antenna coupler for silicon photonics characterized by third-harmonic generation. ACS Photonics.

[33] Vercruysse D., Neutens P., Lagae L., Verellen N., Van Dorpe P. (2017). Single asymmetric plasmonic antenna as a directional coupler to a dielectric waveguide. ACS Photonics.

[34] Ge Z., Zhang L., Wang G., Zhang W., Liu M., Li S., Wang L., Sun Q., Ren W., Si J. (2017). On-chip router elements based on silicon hybrid plasmonic waveguide. IEEE Photonics Technol. Lett..

[35] Wang S., Liu T. (2016). Four-port polarization and topological charge controlled directional plasmonic coupler. IEEE Photonics Technol. Lett..

[36] Guo Y., Pu M., Li X., Ma X., Song S., Zhao Z., Luo X. (2018). Chip-integrated geometric metasurface as a novel platform for directional coupling and polarization sorting by spin–orbit interaction. IEEE J. Sel. Top. Quantum Electron.

[37] Huang L., Chen X., Bai B., Tan Q., Jin G., Zentgraf T., Zhang S., Applications (2013). Helicity dependent directional surface plasmon polariton excitation using a metasurface with interfacial phase discontinuity. Light Sci. Appl..

[38] Pu M., Li X., Ma X., Wang Y., Zhao Z., Wang C., Hu C., Gao P., Huang C., Ren H. (2015). Catenary optics for achromatic generation of perfect optical angular momentum. Sci. Adv..

[39] Li X., Pu M., Zhao Z., Ma X., Jin J., Wang Y., Gao P., Luo X. (2016). Catenary nanostructures as compact Bessel beam generators. Sci. Rep..

[40] Luo X.-G., Pu M.-B., Li X., Ma X.-L. (2017). Broadband spin Hall effect of light in single nanoapertures. Light Sci. Appl..

[41] Guo Y., Pu M., Li X., Ma X., Luo X. (2018). Ultra-broadband spin-controlled directional router based on single optical catenary integrated on silicon waveguide. Appl. Phys. Express.

[42] Clifford T. (1960). The Rational Mechanics of Flexible or Elastic Bodies, 1638–1788.

[43] Heyman J. (1998). Hooke’s cubico–parabolical conoid. Notes Rec. R. Soc. Lond..

[44] Gilbert D. (1826). On the Mathematical Theory of Suspension Bridges, with Tables for Facilitating Their Construction.

[45] Arbabi A., Horie Y., Bagheri M., Faraon A. (2015). Dielectric metasurfaces for complete control of phase and polarization with subwavelength spatial resolution and high transmission. Nat. Nanotechnol..

[46] Meng Y., Hu F., Liu Z., Xie P., Shen Y., Xiao Q., Fu X., Bae S.-H., Gong M. (2019). Chip-integrated metasurface for versatile and multi-wavelength control of light couplings with independent phase and arbitrary polarization. Opt. Express.

[47] Yu N., Genevet P., Aieta F., Kats M.A., Blanchard R., Aoust G., Tetienne J.-P., Gaburro Z., Capasso F. (2013). Flat optics: Controlling wavefronts with optical antenna metasurfaces. IEEE J. Sel. Top. Quantum Electron.

[48] Edward D.P. (1985). Handbook of Optical Constants of Solids.

[49] Johnson P.B., Christy R.-W. (1972). Optical constants of the noble metals. Phys. Rev. B.

[50] Bourke L., Blaikie R.J. (2017). Genetic algorithm optimization of grating coupled near-field interference lithography systems at extreme numerical apertures. J Opt.

[51] Jiang J., Wu H., Jiang L., Li X. (2012). Genetic optimization of double subwavelength metal slits surrounded by surface dielectric gratings for directional beaming manipulation. Opt. Commun..

[52] Shirakawa T., Ishikawa K.L., Suzuki S., Yamada Y., Takahashi H. (2010). Design of binary diffractive microlenses with subwavelength structures using the genetic algorithm. Opt. Express.

